# Therapeutic and Radiosensitizing Effects of Armillaridin on Human Esophageal Cancer Cells

**DOI:** 10.1155/2013/459271

**Published:** 2013-06-24

**Authors:** Chih-Wen Chi, Chien-Chih Chen, Yu-Jen Chen

**Affiliations:** ^1^Department of Medical Research, Mackay Memorial Hospital, Taipei 25160, Taiwan; ^2^Department of Biotechnology, Hungkuang University, Taichung 43302, Taiwan; ^3^Department of Radiation Oncology, Mackay Memorial Hospital, Taipei 10449, Taiwan; ^4^Graduate Institute of Pharmacology, Taipei Medical University, Taipei 11031, Taiwan

## Abstract

*Background*. Armillaridin (AM) is isolated from *Armillaria mellea*. We examined the anticancer activity and radiosensitizing effect on human esophageal cancer cells. *Methods*. Human squamous cell carcinoma (CE81T/VGH and TE-2) and adenocarcinoma (BE-3 and SKGT-4) cell lines were cultured. The MTT assay was used for cell viability. The cell cycle was analyzed using propidium iodide staining. Mitochondrial transmembrane potential was measured by DiOC_6_(3) staining. The colony formation assay was performed for estimation of the radiation surviving fraction. Human CE81T/VGH xenografts were established for evaluation of therapeutic activity *in vivo*. *Results*. AM inhibited the viability of four human esophageal cancer cell lines with an estimated concentration of 50% inhibition (IC_50_) which was 3.4–6.9 *μ*M. AM induced a hypoploid cell population and morphological alterations typical of apoptosis in cells. This apoptosis induction was accompanied by a reduction of mitochondrial transmembrane potential. AM accumulated cell cycle at G_2_/M phase and enhanced the radiosensitivity in CE81T/VGH cells. *In vivo*, AM inhibited the growth of CE81T/VGH xenografts without significant impact on body weight and white blood cell counts. *Conclusion*. Armillaridin could inhibit growth and enhance radiosensitivity of human esophageal cancer cells. There might be potential to integrate AM with radiotherapy for esophageal cancer treatment.

## 1. Introduction

**Figure 1:**
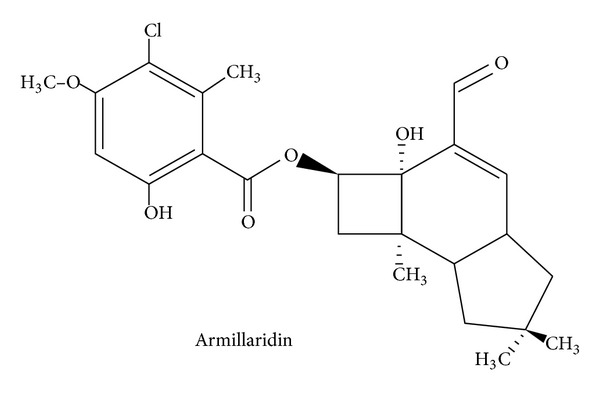
Chemical structure of Armillaridin.

*Armillaria mellea* is a medicinal and edible fungus with a symbiotic relationship with the Chinese medicinal herb *Gastrodia elata* (Tien-Ma). It is commonly used in herbal medicine to treat vertigo, dizziness, headache, numbness in limbs, and infantile convulsions. Extracts of *Armillaria mellea* had been reported to be bioactive in antioxidation [1] and in lymphocyte proliferation [2]. Armillaridin (AM) is a sesquiterpenoid aromatic ester compound isolated from the mycelium of *Armillaria mellea *[3]. It is a colorless, acicular compound with antibacterial activities [3]. The chemical structure is shown in Figure 1.

Esophageal cancer is the malignancy with the worst prognosis, with an average 5-year survival rate less than 25% [4]. Locally advanced esophageal carcinoma is known to be refractory to a single modality of treatment. Patients with unresectable or medically inoperable disease are usually treated with radiation therapy and concurrent chemotherapy [5–7]. Although various chemotherapy regimens are available, esophageal cancer carries a very poor prognosis, with a mean survival time of less than 8.1 months treated with current agents, single or in combination [8]. Clearly, the development of novel and potent compounds to use against the disease or to enhance the radiotherapy efficacy to ameliorate both local and distant tumor control in esophageal cancer is an urgent task.

In the present study, we examined the cytotoxic effects of AM on human esophageal cancer cell lines. The radiosensitizing activity of AM was also tested.

## 2. Materials and Methods

### 2.1. Preparation of Armillaridin and Determination of Purity

AM, 3-chloro-6-hydroxy-4-methoxy-2-methyl-,3-formyl-2,2a,4a,5,6,7,7a,7b-octahydro-2a-hydroxy-6, 6,7b-trimethyl-1H-cyclobut(e)inden-2-yl ester, was isolated from *Armillaria mellea*. It was dissolved in dimethylsulfoxide (DMSO). AM was stored as stock solution at −20°C. The working solution was freshly prepared prior to each experiment. In the cell culture experiments, the final concentration of DMSO was maintained at levels not exceeding 0.1% (v/v), which has been demonstrated to have no influence on cell growth.

### 2.2. Cell Culture

The human squamous cell carcinoma (CE81T/VGH and TE-2) and adenocarcinoma (BE-3 and SKGT-4) cell lines were kindly provided by Professor Hu (Veteran's General Hospital, Taipei, Taiwan) and purchased from ATCC, respectively. Cells were cultured in DMEM (GIBCO, Grand Island, NY, USA) supplemented with NaHCO_3_ (10 mmol/L), HEPES (20 mmol/L), and 10% heat-inactivated fetal calf serum (FCS, Hyclone, Logan, UT, USA) in a humidified 5% CO_2_ incubator to maintain exponential growth. 

### 2.3. Cell Viability

To determine the effect on cell viability, cells were treated with various concentrations of AM. Cell viability was assessed by an MTT (3-(4,5-dimethylthiazol-2-yl)-2,5-diphenyl-tetrazolium bromide) assay. Briefly, 1 mg/mL MTT was added to the culture medium, and the cells were incubated at 37°C for 4 h. An equal volume of acid isopropanol (0.04 M HCl in isopropanol) was added to dissolve the formazan inside the viable cells. The absorbance was measured at 570 nm using an ELISA reader. All experiments for measurement were triplicated. The concentration of 50% inhibition (IC_50_) values was calculated by GraphPad Prism 4 software (San Diego, CA, USA).

### 2.4. Cell Morphology

Cell morphology of CE81T/VGH with 0 and 10 *μ*M AM for 3 days was observed after Liu's staining. The micrographs were taken by 1000x light microscope (Olympus).

### 2.5. Cell Cycle Analysis

Cells treated with AM were harvested and washed with phosphate buffered saline (PBS), then fixed and permeated at 4°C with ethanol. Cells were stained with propidium iodide (PI) solution (PI, 0.5 mg/mL; RNAse, 0.1 mg/mL; Sigma) from a CycleTEST^plus^ DNA reagent kit (Becton Dickinson, Lincoln Park, NJ, USA) in the dark. Analysis of DNA histogram was performed by a FACScalibur flow cytometer (Becton Dickinson, Lincoln Park, NJ, USA). The data from 10^4^ cells were collected and analyzed using ModFit software (Becton Dickinson, Lincoln Park, NJ, USA) to calculate the proportion of cells at G_2_/M phase.

### 2.6. Mitochondrial Membrane Potential

Mitochondrial membrane permeabilization (MMP) was analyzed by the staining of the mitochondrial inner membrane with the lipophilic fluorescence dye, 40 nM 3,3′-dihexyloxacarbocyanine iodide (DiOC_6_(3)) (Molecular Probes, Eugene, OR, USA), and added to cells for 15 minutes at 37°C in a humidified 5% CO_2_ incubator. The cells were analyzed immediately using a FACS Calibur flow cytometer (Becton Dickinson, San Jose, USA) equipped with a standard 15 mW argon-ion laser (488 nm) used to excite DiOC_6_(3), and a narrow band filter was used to collect emissions between 515 nm and 545 nm. A minimum of ten thousand cells were analyzed by flow cytometry for each data point.

### 2.7. Annexin-V and PI Staining for Apoptosis Assessment

To assess apoptosis, cells were stained with Annexin V-fluorescent isothiocyanate (FITC) conjugate and PI. After being AM treated, cells were washed with PBS, then resuspended in 100 *μ*L of Annexin-V-binding buffer, containing 5 *μ*L of FITC-conjugated Annexin-V plus with 10 *μ*L PI (TACS Annexin-V-FITC Apoptosis Detection Kit, R&D Systems), and incubated for 15 min at room temperature. Then, 400 *μ*L of ice cold 1× binding buffer was added followed by fluorocytometric analysis. The data from 10^4^ cells were collected and analyzed using CellQuest Pro Software (Becton Dickinson, Lincoln Park, NJ, USA) to calculate the proportion of cells with early apoptosis.

### 2.8. Caspase Substrate Activity Assay

Caspase activity was measured according to the manufacture of caspase fluorometric substrate set II plus (Medical & Biological Laboratories Co., Ltd., Tokyo, Japan). Cells were pretreated with 20 *μ*M pan caspase inhibitor Z-VAD-FMK (R&D Systems, Inc., Minneapolis, MN, USA) for one hour followed by treatment with AM for 24, 48 hours. Cells were harvested, washed, and counted. Then, the cells were lysed in cell lysis buffer, and protein concentrations were counted by Pierce BCA protein assay kit (Thermo Scientific, IL, USA). Fifty *μ*g cell lysates were mixed with 50 *μ*L of 2× reaction buffer containing DTT, 50 *μ*M of AFC-conjugated caspase substrates. Subsequently, caspase activity was assayed by spectrofluorometer equipped with a 400 nm excitation wavelength and 505 nm emission filter for analysis. The extent of increased caspase activity was defined by a comparison with a vehicle control.

### 2.9. Armillaridin Treatment and Radiation Delivery

Cells were plated onto culture dishes to allow growth in a DMEM medium containing 10% FCS mixed with various concentrations of AM (0, 0.125, 0.25, and 0.5 *μ*M) for 24 hours. Then, the drug was washed out, and the cells were irradiated. Radiation therapy with a 6 MeV electron beam was delivered by a linear accelerator (Clinac 1800, Varian Associates, Inc., Palo Alto, CA, USA) with a dose rate of 2.4 Gy/min at various doses (0, 0.5, 1, 2, 3, and 4 Gy) in a single fraction. The selection of radiation doses depended on our preliminary work on calibration of radiation survival curves of CE81T/VGH cells to ensure adequate coverage from 100% to less than 37% survival (D0 in radiobiology) for further estimation of surviving fraction. For clinical relevance, a radiation dose of 2 Gy was also selected to match the daily fraction size commonly used in clinical practice. Full electron equilibrium was ensured for each fraction by a parallel plate PR-60C ionization chamber (CAPINTEL, Inc., Ramsey, NY, USA).

### 2.10. Clonogenic Assay and Estimation of SER

After radiation, cells were plated for a clonogenic assay. Viable tumor cells (10^3^) were plated into each 35 mm culture dishes and allowed to grow in DMEM containing 10% FCS. After 10–14 days, the culture dishes were stained with 3% crystal violet, and the number of colonies (more than 50 cells) was counted. The mean control plating efficiency for untreated CE81T/VGH HCC cells was around 37%. The surviving fraction was calculated as mean colonies/cells inoculated. Survival curves were fitted by a linear-quadratic model. The sensitizer enhancement ratio (SER) was calculated as the radiation dose needed for radiation alone divided by the dose needed for various concentrations of AM plus radiation at a survival fraction of 37% (D0 in radiobiology).

### 2.11. Animal Model of Esophageal Tumor Xenograft

All experimental protocols involving animals were reviewed and approved by the Institutional Animal Experimentation Committee of Mackay Memorial Hospital. All animal care and husbandry were conducted in accordance with the *Guide for the Care and Use of Laboratory Animals*. After arrival, the animals were kept in our animal facilities for acclimatization for about 7 days, during which time they had free access to food and water. Male nude mice aged 4-5 weeks were obtained from the National Laboratory Animal Center (Taipei, Taiwan) and housed in a rodent facility at 22 ± 1°C with a 12-hour light-dark cycle. 10^6^ CE81T/VGH cancer cells in 0.1 mL PBS were subcutaneously implanted in the right gluteal region. After 21 days, the tumors approximately grew to 0.5 cm in diameter and the animals were subjected to further experiments.

### 2.12. *In Vivo* Therapeutic Studies

Animals were arranged in groups of 5 to 7 mice. AM at a dose of 80 mg/kg was intraperitoneally (I.P.) injected three times per week for 12 doses. Mice treated with equal amounts of vehicle and 2.5 mg/kg cisplatin were used as vehicle and positive controls. The size of the implanted tumor was measured by the same observer. Calipers were used to measure the largest (*a*) and smallest (*b*) diameters, and the tumor volumes were estimated according to the formula 0.5*ab* ^2^. The total body weight of each mouse was determined every other day. The leukocyte count was estimated by retro-orbital blood sampling every other day during the whole study period.

### 2.13. Statistics

Data were presented as mean ± standard error from triplicated experiments. Statistical comparisons were made using Student's *t*-test or one-way analysis of variance (ANOVA) as indicated. The difference was considered significant for *P* < 0.05. Data analysis was performed using SPSS software (version 10.0, Chicago, IL, USA). We used Sigma Plot software (version 8.0, SPSS Inc., Chicago, IL, USA) with syntax to fit survival curves with a linear-quadratic model.

## 3. Results

### 3.1. Cell Viability

AM inhibited the viability of human esophageal cancer cell lines, including squamous cell carcinoma (CE81T/VGH and TE-2) and adenocarcinoma (BE-3 and SKGT-4), in a time- and concentration-dependent manner. The estimated IC_50_ for these four cell lines ranged from 3.4–6.9 *μ*M (Table 1).

### 3.2. Hypoploidy, Mitochondria Transmembranous Potential, and Morphological Alteration

**Figure 2:**
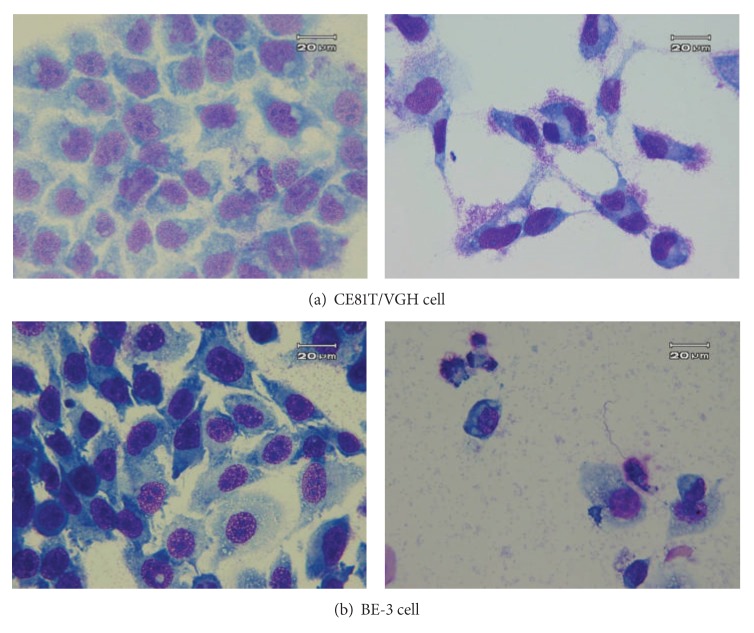
The morphology of esophageal cancer cells treated by Armillaridin. Cells were treated with a vehicle as control (left) or Armillaridin 10 *μ*M for 3 days (right). (a) CE81T/VGH squamous carcinoma cells. (b) BE-3 adenocarcinoma cells.

**Figure 3:**
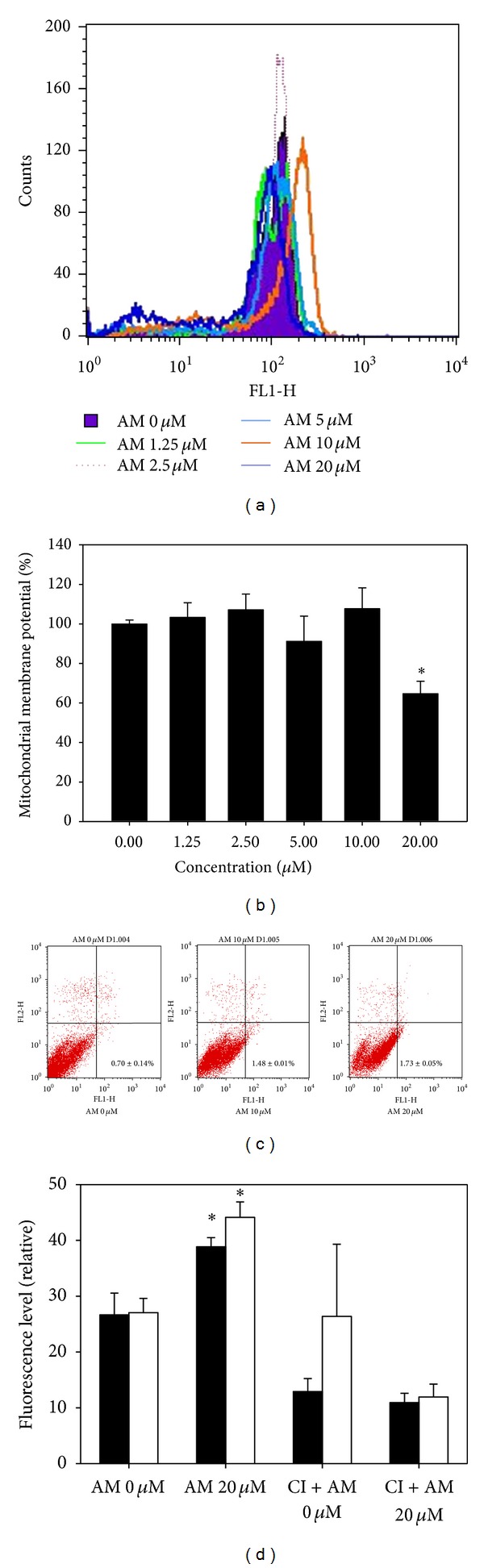
Effect of Armillaridin on mitochondrial transmembrane potential reduction, apoptosis, and caspase inhibitor activity in CE81T/VGH cells. (a) Representative mitochondrial transmembrane potential histograms were shown as CE81T/VGH cells treated with Armillaridin for 24 hours. (b) Relative level of mitochondrial transmembrane potential. (c) The amount of cells with early apoptosis (FITC^+^/PI^−^ cells) by the Annexin V/PI staining assay. (d) Caspase 3 activity with or without pretreatment with pan caspase inhibitor at day 1 (closed column) and day 2 (opened column). Results are mean ± SE from three independent experiments. Significant differences between control cells and cells treated with AM 20 *μ*M are indicated by *P < 0.001. AM: Armillaridin.

Armillaridin induced morphological alterations, such as chromatin condensation, membrane blebbing and apoptotic body formation, which are typical of apoptosis in CE81T/VGH and BE-3 cells (Figure 2). This apoptosis induction activity was accompanied by a significant reduction of mitochondrial transmembrane potential in cells, indicating that an intrinsic apoptotic pathway was involved (Figure 3). The amount of cells with early apoptosis, as shown in proportion of FITC^+^/PI^−^ cells by the Annexin V/PI staining assay, increased after treatment with AM (Figure 3(c)). AM, at concentration-induced extensive apoptosis (20 *μ*M), increased the enzymatic activity of caspase 3, and this increment was blocked by pretreatment with pan caspase inhibitor z-VAD-fmk (Figure 3(d)).

### 3.3. Cell Cycle Analysis

**Figure 4:**
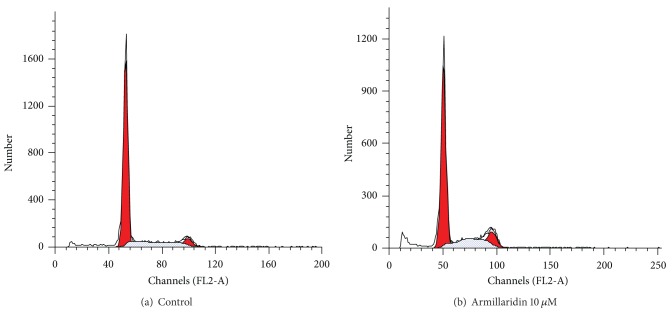
Cell cycle analysis of esophageal cancer cells. DNA histograms were demonstrated for CE81T/VGH cells after 3 days of culture. (a) Control. (b) Armillaridin 10 *μ*M.

As shown in Figure 4, AM treatment caused the development of a hypoploid cell population and an accumulation of cell cycle at G_2_/M phase. The populations of G_2_/M phase of control and AM-treated cells were 5.91 ± 0.43% and 14.04 ± 3.17%, respectively. The percentage of sub-G1 phase was increased from 4.44 ± 0.01% of control to 8.72 ± 2.47% of AM-treated cells.

### 3.4. Growth Inhibitory Effect on Tumor Xenograft

**Figure 5:**
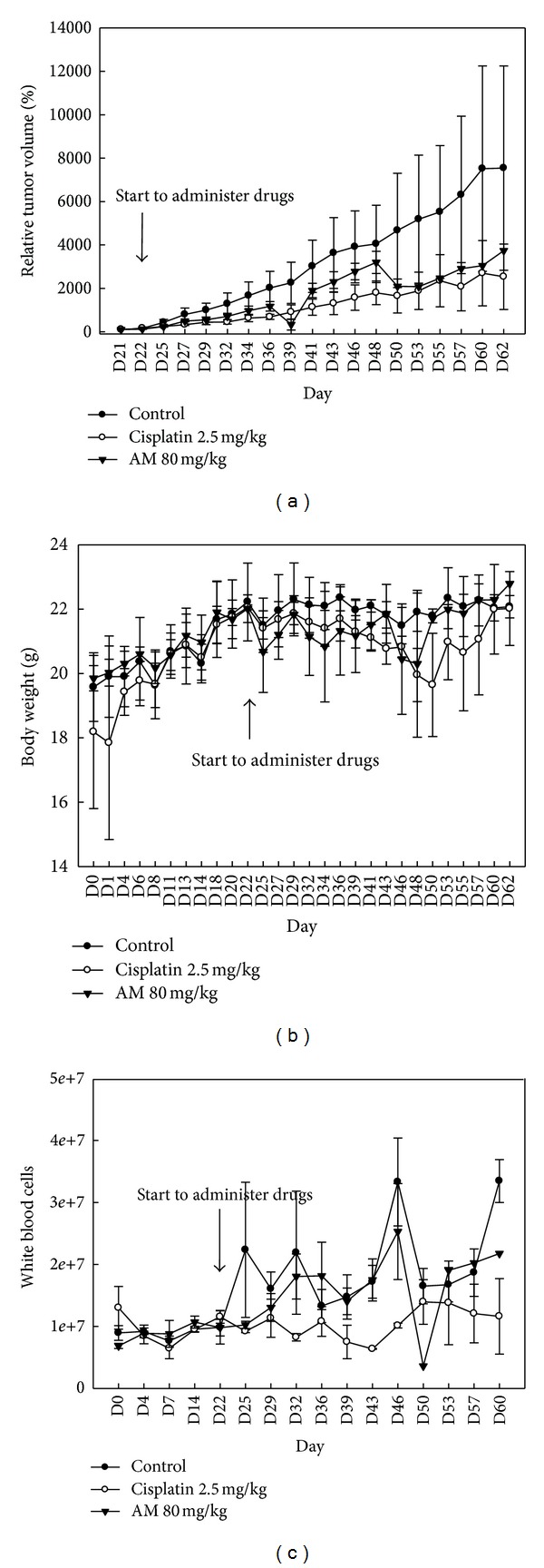
Armillaridin inhibited the growth of esophageal cancer xenograft. Balb/c nude mice at 6 weeks of age were subcutaneously injected with 10^6^ CE81T/VGH cells at day 0. Treatments were started on day 22 with triple administration per week for 12 doses. Data presented are mean ± SE (*n* = 3/group). The tumor size observed in the Armillaridin-treated group (80 mg/kg) was significantly decreased than in vehicle-treated group (a). There are no significant differences in body weight growth kinetic between control and Armillaridin-treated groups (b). No marked effect was observed in the white blood cell counts (c). AM: Armillaridin.

AM inhibited the growth of CE81T/VGH xenografts without significant impact on body weight and white blood cell counts (Figure 5).

### 3.5. Radiosensitizing Activity *In Vitro*

**Figure 6:**
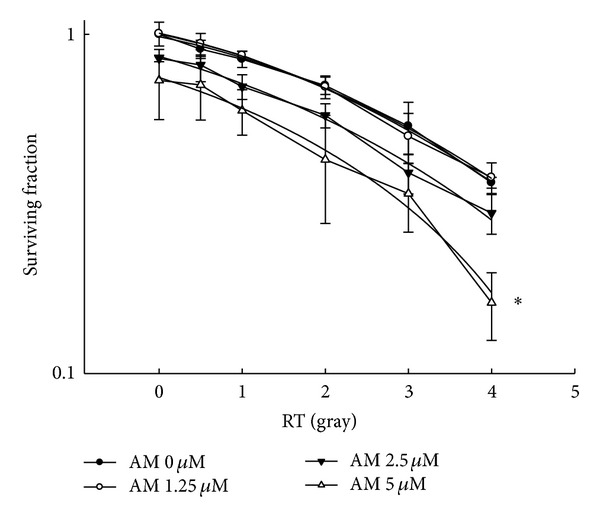
Armillaridin enhanced radiosensitivity of human esophageal CE81T/VGH cancer cells. CE81T/VGH cells were treated with vehicle (closed circle), AM 1.25 *μ*M (opened circle), 2.5 *μ*M (closed inverted triangle), or 5 *μ*M (opened triangle) for 24 h before radiation. After radiation, drugs were washed out, and cells were cultured for 14 days for colony formation assay. Data from three separate experiments are expressed as mean ± SE. Significant differences between control cells and cells treated with AM 5 *μ*M are indicated by *P < 0.05.

AM at 2.5 and 5.0 *μ*M enhanced the radiosensitivity of CE81T/VGH cells with a sensitizer enhancement ratio up to 1.6 (Figure 6).

## 4. Discussion

The need of novel therapeutics against esophageal cancer remains great in current clinical practice. We found that AM, a natural occurring compound isolated from the medicinal fungus *Armillaria mellea*, possesses activity that inhibits growth and enhances the radiosensitivity of human esophageal cancer cells. The therapeutic potential of this compound was ascertained by the current standard of care using concurrent chemoradiation for locally advanced esophageal cancer.

The results of this study demonstrated that the mode of AM-induced cell death might be apoptosis. A mitochondrial pathway was postulated for apoptosis caused by AM in human esophageal cancer cells. Results of caspase substrate and pan caspase inhibitor assays suggest that caspase 3 might be one of the targets of AM in this experimental model. *In vivo* xenograft experiments showed moderate tumor inhibitory activity against human esophageal cancer with a relative safety profile in comparison with the standard first-line chemotherapeutic agent cisplatin. Although cisplatin has been used as a recommended chemoradiation regimen, the morbidity additive to radiotherapy toxicity remains the major drawback in clinical application [9, 10]. For combination with radiation therapy, the drugs possessing characteristics of moderate tumor cytotoxicity without major toxicity are regarded as viable candidates. Taken together, AM could be considered a novel agent for combination with radiotherapy to treat esophageal cancer.

To enhance the radiotherapy efficacy against cancer, several strategies have been utilized. Modulation of DNA damage repair [11], cellular antioxidant machinery [12], prosurvival signaling [13], tumor hypoxia state [14], and cell cycle distribution [15] are commonly used targets for the development of radiation sensitizers. In the present study, AM was shown to increase the percentage of cells at the G_2_/M phase. Given that cells at the G_2_/M phase are most sensitive to radiation [16], we further examined the radiosensitizing activity of AM and noted a sensitizer enhancement ratio up to 1.6. In clinical radiotherapy for esophageal cancer, the major concern is for the normal lung, spinal cord, and heart tissues near the esophageal tumor. By using a radiosensitizer, such as AM, the needed radiation dose for a tumor might be reduced, and, thus, the normal tissue injury could be decreased simultaneously. This therapeutic application of AM remains to be validated by *in vivo* experiments and to be optimized for combinatory conditions with radiation therapy.

In conclusion, the medicinal fungus component Armillaridin is capable of inhibiting growth and enhancing the radiosensitivity of human esophageal cancer cells.

## Figures and Tables

**Table 1:** The half maximal inhibitory concentration (IC_50_) of Armillaridin on various human esophageal cancer cell lines.

Cell lines	IC_50_
Squamous cell carcinoma	
CE81T/VGH	6.9 *μ*M
TE-2	3.4 *μ*M
Adenocarcinoma	
BE-3	5.4 *μ*M
SKGT-4	5.5 *μ*M

## References

[1] Lung M-Y, Chang Y-C (2011). Antioxidant properties of the edible basidiomycete Armillaria mellea in submerged cultures. *International Journal of Molecular Sciences*.

[2] Sun Y, Liang H, Zhang X, Tong H, Liu J (2009). Structural elucidation and immunological activity of a polysaccharide from the fruiting body of Armillaria mellea. *Bioresource Technology*.

[3] Junshan Y, Yuwu C, Xiaozhang F (1984). Chemical constituents of Armillaria mellea mycelium I. Isolation and characterization of armillarin and armillaridin. *Planta Medica*.

[4] Jemal A, Siegel R, Ward E, Hao Y, Xu J, Thun MJ (2009). Cancer statistics, 2009. *CA Cancer Journal for Clinicians*.

[5] Cooper JS, Guo MD, Herskovic A (1999). Chemoradiotherapy of locally advanced esophageal cancer: long-term follow-up of a prospective randomized trial (RTOG 85-01). *Journal of the American Medical Association*.

[6] Tepper J, Krasna MJ, Niedzwiecki D (2008). Phase III trial of trimodality therapy with cisplatin, fluorouracil, radiotherapy, and surgery compared with surgery alone for esophageal cancer: CALGB 9781. *Journal of Clinical Oncology*.

[7] Liu H-C, Hung S-K, Huang C-J (2005). Esophagectomy for locally advanced esophageal cancer, followed by chemoradiotherapy and adjuvant chemotherapy. *World Journal of Gastroenterology*.

[8] Chiarion-Sileni V, Corti L, Ruol A (2007). Phase II trial of docetaxel, cisplatin and fluorouracil followed by carboplatin and radiotherapy in locally advanced oesophageal cancer. *British Journal of Cancer*.

[9] Herskovic A, Martz K, Al-Sarraf M (1992). Combined chemotherapy and radiotherapy compared with radiotherapy alone in patients with cancer of the esophagus. *The New England Journal of Medicine*.

[10] Kuwahara A, Yamamori M, Nishiguchi K (2009). Replacement of cisplatin with nedaplatin in a definitive 5-fluorouracil/cisplatin-based chemoradiotherapy in Japanese patients with esophageal squamous cell carcinoma. *International Journal of Medical Sciences*.

[11] Kesari S, Advani SJ, Lawson JD (2011). DNA damage response and repair: insights into strategies for radiation sensitization of gliomas. *Future Oncology*.

[12] Pajonk F, Vlashi E, McBride WH (2010). Radiation resistance of cancer stem cells: the 4 R’s of radiobiology revisited. *Stem Cells*.

[13] Mokim Ahmed K, Li JJ (2008). NF-*κ*B-mediated adaptive resistance to ionizing radiation. *Free Radical Biology and Medicine*.

[14] Brizel DM, Sibley GS, Prosnitz LR, Scher RL, Dewhirst MW (1997). Tumor hypoxia adversely affects the prognosis of carcinoma of the head and neck. *International Journal of Radiation Oncology Biology Physics*.

[15] Pawlik TM, Keyomarsi K (2004). Role of cell cycle in mediating sensitivity to radiotherapy. *International Journal of Radiation Oncology Biology Physics*.

[16] Sinclair WK, Morton RA (1966). X-ray sensitivity during the cell generation cycle of cultured Chinese hamster cells. *Radiation Research*.

